# Prospective cohort study of parathyroid function and quality of life after total thyroidectomy for thyroid cancer: robotic surgery vs. open surgery

**DOI:** 10.1097/JS9.0000000000000725

**Published:** 2023-09-26

**Authors:** Xiangquan Qin, Jia Luo, Jing Ma, Xiaozheng Cao, Jinjin Zhao, Jun Jiang, Yi Zhang, Lingjuan Zeng, Linjun Fan

**Affiliations:** Department of Breast and Thyroid Surgery, Southwest Hospital, the First Affiliated Hospital of the Army Military Medical University, Gaotanyan Street 29, Shapingba District, Chongqing, 400038, China

**Keywords:** hypoparathyroidism, quality of life, robot surgery, super-meticulous capsular dissection (SMCD), thyroidectomy

## Abstract

**Objective:**

To compare robot-assisted thyroidectomy (RT) and open thyroidectomy (OT) through a prospective cohort study focusing on the rate of postoperative hypoparathyroidism, efficacy, and quality of life (QoL).

**Summary background data:**

Hypoparathyroidism is a frequent complication after thyroidectomy. Reducing the risk of hypoparathyroidism after total thyroidectomy is a crucial and difficult task for thyroid surgeons.

**Methods:**

We prospectively enroled 306 patients with papillary thyroid carcinoma into an RT group and OT group. The former used “super-meticulous” capsular dissection) and the latter used traditional meticulous capsular dissection. Patients were evaluated by scales [Short Form (SF)-36, Visual Impairment Scale (VIS), Swallowing Impairment Scale (SIS), Neck Impairment Scale (NIS), Scar questionnaire (SCAR-Q)].

**Results:**

The rates of transient hypoparathyroidism, permanent hypoparathyroidism, and transient hypocalcemia after surgery in the OT group and RT group were significantly different (*P*<0.001). SIS and VIS scores in the two groups were significantly different (*P*<0.001). SF-36 showed significant differences (*P*<0.001) in the subsections of “physiological function”, “body pain”, “general health”, “vitality”, “social function”, “role emotional”, and “mental health” between the two groups. SCAR-Q showed that the length and appearance of scars showed significant differences between the two groups.

**Conclusions:**

RT with Super-meticulous capsular dissection can protect parathyroid function and improve postoperative QoL, and could be a new option for robot-assisted surgery against thyroid cancer.

## Introduction

Highlights

- We prospectively enroled 306 patients with differentiated thyroid cancer into a robot-assisted thyroidectomy group and an open thyroidectomy group, and evaluated the rate of postoperative hypoparathyroidism, efficacy, and quality of life. Overall, robot-assisted thyroidectomy with super-meticulous capsular dissection can protect parathyroid function and improve postoperative quality of life.

Thyroid cancer (TC) has a high incidence, and an increasing number of young individuals are suffering from it^1^. Neck scars and related complications resulting from conventional open thyroidectomy (OT) can affect the postoperative quality of life (QoL) of patients^2^.

As a common complication after thyroidectomy, hypoparathyroidism is caused by intraoperative injury to the blood supply^3^ or inadvertent removal of the parathyroid glands (PGs)^4^. Permanent hypoparathyroidism often leads to impaired postoperative QoL, which is often seriously underestimated^5^. Preservation of the PGs *in situ* and their blood supply is a highly challenging procedure during thyroidectomy.

Some methods are available to identify and protect PGs during thyroidectomy: (1) nano-carbon negative PG imaging is used intraoperatively, but this method is only helpful for identifying PGs. Intraoperative detection of PG autofluorescence is another method for identifying and protecting PGs^6,7^, but the current application of this technique is relatively limited, so its promotion is restricted^8,9^; (2) meticulous capsular dissection (MCD) is often used to remove the thyroid gland as close as possible to the true thyroid capsule to prevent unintentional removal of PGs^10^. However, the recognition ability of the naked eye and conventional instruments often limit visibility and increase the risk of damaging the blood supply to A1 and A2 PGs. Retention of the subcapsular PGs (A3 type) in situ is almost imppossibe with MCD^11^. The rates of transient hypoparathyroidism and permanent hypoparathyroidism can reach 51.9% and 16.2%, respectively, if the aforementioned methods are employed for PG protection during total thyroidectomy and dissection of central lymph nodes^11^.

Reducing the incidence of hypoparathyroidism after thyroidectomy is an urgent problem. To improve the effect of thyroidectomy, our team has developed an innovative “super-meticulous” capsular dissection (SMCD) method for robotic surgery after early exploration^12^. We wished to observe and validate the advantages of SMCD in PG protection. We compared robot-assisted thyroidectomy (RT) and OT through a prospective cohort study focusing on the rate of postoperative hypoparathyroidism, efficacy, and QoL.

## Methods

The work has been reported in line with the STROCSS criteria^13^.

### Patient selection and study design

**Figure 1:**
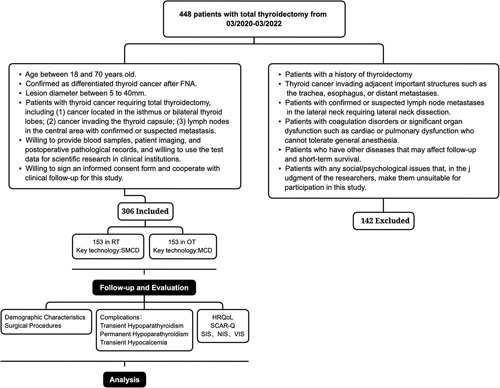
Technology roadmap. HRQoL, health-related quality of life; OT, open thyroidectomy; MCD, meticulous capsular dissection; NIS, Neck Impairment Score; SCAR-Q, Scar questionnaire; SIS, Swallowing Impairment Score; VIS, Voice impairment Score.

From March 2020 to March 2022, we enroled 448 patients with differentiated PTC necessitating total thyroidectomy. The advantages and disadvantages of RT and OT were explained fully to patients, and the corresponding surgical method was selected based on their preference. Ultimately, 306 patients in two groups were willing to be enroled and received postoperative follow-up. The rates of postoperative hypoparathyroidism and other complications, the levels of postoperative on-Tg and on-TgAb, and the number of cases who received ^131^I treatment were evaluated. In addition, all patients completed assessment using the Short From (SF)-36 scale^5^, Voice Impairment Score (VIS)^14^, Swallowing Impairment Score (SIS), Neck Impairment Score (NIS)^15^, and Scar questionnaire (SCAR-Q) scale^16,17^. Surgical costs, total hospitalization costs, and other aspects were also analyzed statistically for all patients (Fig. 1).

### Procedures

Both surgical procedures involved the use of endotracheal intubation, general anaesthesia, and neck hyperextension, as well as routine disinfection and draping of the surgical site. Moreover, intraoperative neural monitoring was used in both surgical procedures to examine the recurrent laryngeal nerve^18^.

#### Open thyroidectomy

We have outlined extensively the operative procedure of OT previously^19^. Herein, we focused on MCD. During thyroidectomy, the dissection and detachment of the posterior thyroid gland were undertaken close to the true capsule. The RLN was exposed fully and protected throughout the posterior and central regions of the thyroid gland. Preservation of all PGs and their blood supply was prioritized whenever possible. If a PG was at risk of ischaemia or mis-incised, it was minced and injected into the ipsilateral sternocleidomastoid muscle.

#### Robot-assisted thyroidectomy

**Figure 2:**
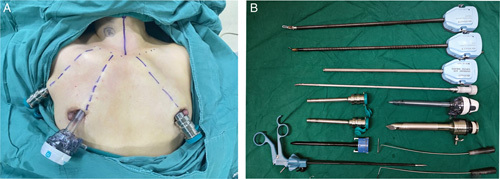
(A) Patient position. (B) Surgical instruments.

Previously, we provided a comprehensive introduction to the operative process of RT^19^. RT involves establishing a working space, flap dissection, removal of the thyroid gland, and dissection of central lymph nodes. Herein, we focused on detailing the SMCD procedure for PG preservation *in situ* via a unilateral axilla-bilateral areola (UABA) approach using the example of right-lobe removal (Fig. 2A,B).

**Figure 3:**
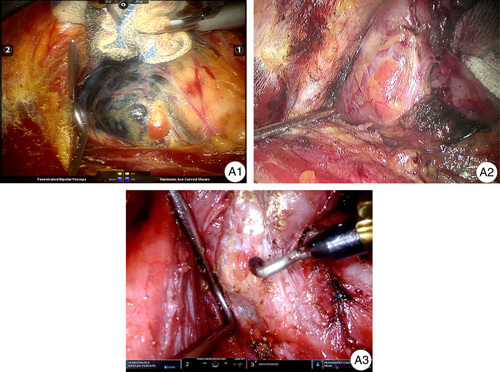
Classification of parathyroid. Type A1 parathyroid glands are located on the surface of the thyroid gland, adhering to its outer layer. Type A2 parathyroid glands are partially or entirely embedded within the thyroid gland, but they remain outside the thyroid’s true capsule. Type A3 parathyroid glands are entirely enclosed within the thyroid's true capsule, representing an intracapsular location.

SMCD was used to incise the true capsule at the back of the thyroid gland. The capsule was peeled off completely from the bottom to top. If the lesion invaded the thyroid capsule, priority was given to lesion resection without retention of the true capsule. PGs, the confluence of the inferior and superior thyroid arteries (secondary vessels), and the tiny branches (tertiary vessels) entering PGs were preserved together. Gradual coagulation and closure of the vascular branches between the true capsule and parenchyma of the thyroid gland were undertaken against the thyroid gland, and preservation of all PGs and all their vascular components was carried out (Fig. 3). A safe distance (>2 mm) to the RLN was needed when freeing and cutting the berry ligament at the entrance of the RLN into the larynx^14^. This method was effective in preserving compact A1 (Video 1) and embedded A2 (Video 2) PGs. Due to the incision and preservation of the true capsule, the A3 PG located below the true capsule could also be identified and preserved (Video 3).

### Complications

Episodes of transient hypoparathyroidism, permanent hypoparathyroidism^20^, postoperative on-Tg and on-TgAb 6 months after surgery^21^, temporary and permanent hoarseness^22^, and postoperative bleeding^23^ were documented (Tables 1 – 3).

**Table 1:** Demographic characteristics of patients, surgical procedures, and perioperative characteristics.

	OT group (*N* = 153)	RT group (*N* = 153)	*P*
Age,years			<0.001
18–35	29 (19.0)	62 (40.5)	
>35	124 (81.0)	91 (59.5)	
Sex			0.89
Female	116 (75.8)	118 (77.1)	
Male	37 (24.2)	35 (22.9)	
Ethnicity			0.24
Han	141 (92.2)	146 (95.4)	
Other	12 (7.8)	7 (4.6)	
Marital status			0.25
Unmarried	7 (4.6)	15 (9.8)	
Married	142 (92.7)	135 (88.2)	
Divorced	3 (2.0)	3 (2.0)	
Widow	1 (0.7)	0	
BMI (kg/m^2^)			0.93
<25	101 (66.0)	104 (68.0)	
25–30	44 (28.8)	41 (26.8)	
>30	8 (5.2)	8 (5.2)	
Comorbidity			
Hypertension	17 (11.1)	18 (11.8)	0.86
Diabetes mellitus	6 (3.9)	4 (2.6)	0.52
Smoker			1
Yes	16 (10.5)	16 (10.5)	
No	137 (89.5)	137 (89.5)	
Alcohol consumption			0.07
Yes	15 (9.8)	26 (17.0)	
No	138 (90.2)	127 (83.0)	
Tumour diameter by sonography (mm)	10.18±3.71	9.46±3.97	0.1
Tumescent-neck lymph nodes			0.06
Yes	22 (13.8)	34 (22.2)	
No	131 (86.2)	119 (77.8)	
T			
T1 (<2 cm)	150 (98.0)	150 (98.0)	1
T2 (2–4 cm)	3 (2.0)	3 (2.0)	
N			0.08
N0	131 (85.6)	119 (77.8)	
N1	22 (14.4)	34 (22.2)	
Tumour location			0.03
Unilateral	101 (66.0)	120 (78.4)	
Bilateral	47 (30.7)	32 (20.9)	
Isthmus	5 (3.3)	1 (0.7)	
Physical examination (degree)			0.32
0	132 (86.5)	126 (82.4)	
I	21 (13.5)	27 (17.6)	
Gene-mutation (BRAF)			0.93
NA	46 (30.1)	43 (28.1)	
Yes	95 (62.1)	98 (64.1)	
No	12 (7.8)	12 (7.8)	
Extracapsular invasion			0.32
Yes	1 (0.70)	0	
No	152 (99.3)	153 (100.0)	
Operative duration (min)			
TT + UCLN	118.34±24.47	119.30±29.17	0.82
TT + BCLN	131.53±28.9	131.43±24.17	0.98
Operation type			0.03
TT + UCLN	95 (62.1)	76 (49.7)	
TT + BCLN	58 (37.9)	77 (50.3)	
Mis-resection of parathyroid glands			<0.001
Yes	56 (36.6)	2 (1.3)	
No	97 (63.4)	151 (98.7)	
No. parathyroid glands transplanted			<0.001
0	101 (66.0)	151 (98.7)	
1	46 (30.1)	1 (0.7)	
2	6 (3.9)	1 (0.7)	
Estimated blood loss (ml)	27.58±16.29	26.60±16.98	0.61
Total number of days of drain insertion^c^	4.02±1.22	4.08±1.3	0.65
Hospitalization duration, days	7.07±1.44	7.35 ±1.55	0.10
Postoperative on-Tg (ng/ml)^a^	1.43±7.54	1.32±4.32	0.89
Postoperative on-TgAb (ng/ml)^a^	52.19±153.84	40.00±117.203	0.59
^131^I therapy^b^			0.11
Yes	55 (35.9)	42 (27.5)	
No	98 (64.1)	111 (72.5)	
Recurrence	0	0	
Metastasis	0	0	
Mean follow-up (months)	30 (14, 39)	29 (14, 40)	0.33

Data are the mean±SD, number (%) and the follow-up time was reported as the median (min, max).

TT + UCLN: Total thyroidectomy + unilateral central lymph node dissection.

TT + BCLN: Total thyroidectomy + bilateral central lymph nodes dissection.

NA, not applicable; OT, open thyroidectomy; RT, robot-assisted thyroidectomy; TT+BCLN, total thyroidectomy + bilateral central lymph nodes dissection; TT+UCLN, total thyroidectomy + unilateral central lymph node dissection.

^a^Postoperative on-Tg and on-TgAb are defined as the Tg and TgAb levels under non-thyroid-stimulated hormone-stimulation on the 6 months after surgery.

^b131^I therapy was defined as patients who had been evaluated by the nuclear-medicine department or confirmed to have a residual thyroid lesion or tumour lesion through ^131^I and required ^131^I therapy on the 6 months after surgery.

^c^Removing the drainage tube is when the drainage volume is below 20 ml.

**Table 2:** Postoperative complications.

	OT group (*N* = 153)	RT group (*N* = 153)	*P*
Haematoma (observed)	1 (0.7)	0	0.32
Tetany	3 (2.0)	0	0.08
Transient hypoparathyroidism^a^	87 (56.9)	20 (13.1)	<0.001
Permanent hypoparathyroidism^a^	9 (5.9)	2 (1.3)	0.03
Temporary hoarseness^b^	17 (11.1)	4 (2.6)	0.003
Permanent hoarseness^b^	2 (1.3)	0	0.16
Lymphorrhagia	1 (0.7)	0	0.32
Postoperative infection	0	1 (0.7)	0.32
Transient hypocalcemia	110 (71.9)	78 (51.0)	<0.001
Postoperative bleeding^c^	2 (1.3)	1 (0.7)	0.56

Data are presented as numbers (%).

OT, open thyroidectomy; RT, robot-assisted thyroidectomy.

^a^“Transient hypoparathyroidism” is defined as a serum PTH level that falls below the normal range on the first day after surgery. However, if the PTH level fails to recover within 6 months after surgery, it is considered to denote permanent hypoparathyroidism.

^b^“Temporary hoarseness” refers to a voice change on the first day after surgery due to injury to the recurrent laryngeal nerve. If hoarseness does not fully recover within 6 months after surgery and is confirmed by laryngoscopy as vocal-cord paralysis, it is defined as “permanent injury to the recurrent laryngeal nerve”.

^c^“Postoperative bleeding” refers to bleeding in the original surgical field or subcutaneous tunnel area that requires further surgical intervention to control the bleeding or clear any subcutaneous haematomas.

**Table 3:** Univariate and multivariate analysis of permanent hypoparathyroidism.

	Univariate		Multivariate	
	HR (95% CI)	*P*	HR (95% CI)	*P*
Age (year)	1.03 (0.97, 1.08)	0.36		
Sex	0.38 (0.05, 3.01)	0.36		
BMI (kg/m^2^)	0.97 (0.81, 1.17)	0.75		
Tumour diameter according to sonography (mm)	1.03 (0.88, 1.20)	0.7		
Tumescent-neck lymph nodes	0.38 (0.08, 1.84)	0.23		
Operation type	1.07 (0.31, 3.75)	0.91		
Transplantation of parathyroid glands	5.96 (1.67, 21.25)	0.006	4.41 (1.16, 16.71)	0.03
Total number of days of drain insertion	1.18 (0.73, 1.91)	0.5		
Postoperative on-Tg (ng/ml)	0.85 (0.57, 1.27)	0.44		
Postoperative on-TgAb (ng/ml)	1.00 (0.99, 1.0)	0.9		
Group	0.23 (0.05, 1.08)	0.06		

HR, hazard ratio; NA, not applicable.

### Evaluation of aesthetic outcomes and health-related quality of life (HRQoL)

HRQoL was assessed in patients with SF-36. The latter is a widely used health-survey tool to evaluate HRQoL^5^. SCAR-Q^16^ includes assessment of appearance, symptoms, and psychosocial parameters^17^ (Table 4).

**Table 4:** Assessment of health-related quality of life (HRQoL).

Outcomes	OT group (*N* = 153)	RT group (*N* = 153)	*P*
SIS^a^	7.05±2.18	6.07±0.36	<0.001
VIS^b^	11.28±3.29	10.23±0.77	<0.001
NIS^c^	10.35±2.69	10.01±0.11	0.12
PF^d^	95.26±5.31	97.76±3.77	<0.001
RF^d^	90.29±24.19	92.27±8.69	0.34
BP^d^	86.18±8.51	89.05±9.36	0.006
GH^d^	58.61±10.68	86.37±14.25	<0.001
VT^d^	75.90±12.09	89.24±10.54	<0.001
SF^d^	87.55±15.96	91.18±13.37	0.032
RE^d^	94.73±15.92	99.56±5.41	<0.001
MH^d^	76.25±14.71	95.52±6.1	<0.001
Scar length (cm)	8.28±2.87	1.34±0.97	<0.001
Obvious scar, *N* (%)			
Yes	54 (35.29)	8 (5.23)	<0.001
No	94 (61.44)	135 (88.23)	
NA	5 (3.27)	10 (6.54)	
SCAR-Q Scale^e^			
Appearance	36.55±9.06	13.39±11.51	<0.001
Symptom	8.18±11.89	6.42±9.53	0.17
Psychosocial	3.50±10.07	2.83±9.22	0.55

BP, body pain; GH, general health; MH, mental health; NIS, Neck Impairment Score; PF, physiological function; RE, role emotional; RF, role physical function; SF, social function; SIS, Swallowing Impairment Score; VIS, Voice impairment Score; VT, vitality.

^a^The SIS is a clinical-assessment tool that evaluates the severity of swallowing disorders or dysphasia. It measures difficulty initiating the swallow, trouble swallowing solids and liquids, and coughing or choking while eating. The score is based on a numerical scale, with higher scores indicating greater degrees of swallowing impairment.

^b^The VIS is a standardized assessment tool used to evaluate voice disorders such as hoarseness, voice changes, and difficulty in speaking. The VIS measures various aspects of voice function (e.g. pitch, loudness, quality, and endurance) and assigns a numerical score to each.

^c^The NIS is a validated assessment tool that quantifies the degree of neck pain and functional disability in individuals with neck pain. The NIS is determined by a series of questions regarding pain and disability associated with daily activities, such as work, recreation, and sleep. Scores range from 0 to 40, with higher scores indicating greater pain and disability in the neck.

^d^Consists of 36 questions that cover eight domains: PF, RF, BP, GH, VT, SF, RE, and MH.

^e^Appearance of scars: this scale measures the appearance of scars in terms of length, width, colour (including how closely it matches skin colour), shape, and size. Adverse symptoms: this scale measures how scars feel with items such as how sore, painful, tight, itchy, or tingly scars feel. Psychosocial impact: this scale measures the psychosocial distress caused by scars, with statements that ask about covering or hiding scars and feeling self-conscious, embarrassed, or upset about scars.

#### Impairment assessment

Impairment was assessed using the NIS, SIS, and VIS^24^ (Table 4).

### Statistical analyses

Continuous variables are summarized as the mean and SD. The duration of follow-up was reported as the median (min, max).Categorical variables are presented as frequencies and proportions. Data from HRqoL and NIS, VIS, SIS, and SCAR-Q were transformed into total scale scores. Univariate and multivariate Cox proportional hazard regression models were implemented to evaluate the influence of variables on permanent hypoparathyroidism. *P* less than 0.05 (two-tailed) was considered significant. Statistical analyses were carried out using SPSS 27 (IBM). Videos for operative procedures were generated using Final Cut Pro 2022 (Apple). Figures for operative procedures were generated using Photoshop 2022 (Adobe).

## Results

### Patient characteristics

We enroled 153 patients in each group after screening. The median duration of follow-up was 30 months for the RT group and 29 months for OT group (*P*=0.33). The number of patients over 35 years of age was 124 (81%) in the OT group and 91 (59.5%) in the RT group, and this difference was significant (*P*<0.001). With regard to tumour-node-metastasis stage, there was no significant difference in the number of people in the T stage and N between the two groups (*P*>0.05). There were no significant differences with respect to tumour diameter, the number of cases with tumescent lymph nodes in the neck, rates of extracapsular invasion and *BRAF* mutation, or the number of cases who received ^131^I therapy between the two groups (*P*>0.05).

### Operative and hospitalization data

The duration of the procedure was similar in the two groups: total thyroidectomy + unilateral central lymph node dissection (TT + UCLN; *P*=0.82) and total thyroidectomy + bilateral central lymph nodes dissection (TT + BCLN; *P*=0.98). The operation types in the two groups were different. There were more patients who underwent TT + BCLN in the RT group than in the OT group (77 *vs.* 53, *P*=0.03). Detailed can be found in Table 1. Besides, mis-resection of PGs was more common in the OT group than in the RT group (56 vs. 1, *P*<0.001). There were no significant differences in the total number of days of drain insertion, postoperative on-Tg, or postoperative on-TgAb between the two groups (*P*>0.05). Besides, there was no case of recurrence or metastasis in either group (Table 1).

### Complications

The rates of transient hypoparathyroidism and permanent hypoparathyroidism in OT group and RT group were [87 (56.90%) vs. 20 (13.10%), *P*<0.001] and [9 (5.90%) vs. 2 (1.30%), *P*=0.03], respectively. Compared with the RT group, the OT group had higher rates of transient hypocalcemia [110 (71.90%) vs. 78 (51.00%), *P*<0.001], temporary hoarseness [17 (11.10%) vs. 4 (2.60%), *P*=0.003], and permanent hoarseness [2 (1.30%) vs. 0, *P*=0.32]. A few cases of haematoma, tetany, and lymphorrhagia occurred in the OT group (Table 2).

We evaluated the prognostic value of permanent hypoparathyroidism by univariate and multivariate Cox proportional hazards analysis. PG transplantation significantly increased the risk of permanent hypoparathyroidism after surgery (hazard ratio = 5.96, 95% CI = 1.67–21.25, *P*=0.006; hazard ratio = 4.41, 95% CI = 1.16–16.72, *P*=0.03). There were no significant differences in the two groups regarding age, sex, body mass index, tumour diameter (as judged by sonography), or operation type (Table 3).

### QoL assessment

#### Impairment

The SIS and VIS in the OT group were higher than those in the RT group (7.05±2.18 vs. 6.07±0.36, *P*<0.001; 11.28±3.29 vs. 10.23±0.77, *P*<0.001).

#### Health-related quality of life

The results for “physiological function”, “general health”, “vitality”, “social function”, “role emotional”, and “mental health” in the RT group were higher than those in the OT group (*P*<0.001). In the “body pain” evaluation, the result for the RT group was significantly higher than that of the OT group (89.05±9.36 vs. 86.18±8.51, *P*=0.006).

#### Aesthetic outcomes

The scar was shorter in the RT group (1.34±0.97 vs. 8.28±2.87 cm, *P*<0.001), and the Scar-Q score was significantly higher in the OT group (36.55±9.06 vs. 13.39±11.51, *P*<0.001). There were no significant differences between the two groups in terms of scar-related symptoms and postoperative psychological effects (*P*>0.05) (Table 4).

## Discussion

Robotic thyroid surgery leads to good cosmetic effects, and the application of robotic technology to thyroidectomy could overcome the limitations of conventional endoscopic surgery^25^. However, its advantages in the efficacy and rate of complications compared with open surgery remains unknown^26^. Previously, our retrospective study demonstrated that robotic surgery had equivalent efficacy and a lower rate of complications than OT^19,27^.

We conducted a prospective cohort study to verify the effect of SMCD in robot-assisted thyroidectomy. Our results showed that RT with SMCD achieved the same curative effect as OT, and provided better protection of PG function, reduced swallowing and voice impairments, and improved QoL and the cosmetic appearance.

Hypoparathyroidism is one of the major complications of TC surgery. Our previous retrospective study showed that robotic surgery had obvious advantages for PG protection compared with open surgery^16^. The present prospective study validated this conclusion. The rates of transient hypoparathyroidism, permanent hypoparathyroidism, and transient hypocalcemia in the RT group were significantly lower than those in the OT group. PG transplantation is considered to be a remedy after mis-resection of PGs. Some guidelines have suggested that strategic transplantation of one PG can reduce (or even avoid) the occurrence of severe permanent hypoparathyroidism^9,28^. However, use of univariate and multivariate analysis after the Cox proportional hazards test identified PG transplantation to be a risk factor for permanent hypoparathyroidism in RT and OT, a conclusion that is consistent with that in previous reports^29^.

Some doubts have been raised about the efficacy of robot-assisted surgery^30^. However, we found robot-assisted thyroidectomy with SMCD via a UABA approach could preserve almost all types of PGs *in situ* (Video 1–3), thereby reducing the rate of postoperative hypoparathyroidism significantly. Hence, SMCD was more effective than traditional MCD for PG preservation *in situ*. The present study showed no significant differences in postoperative on-Tg, postoperative on-TgAb, or the number of patients receiving ^131^I therapy between the two groups, which also indicated that robotic surgery could achieve the same efficacy as open surgery. Some authors have reported no significant difference when comparing the operative time between patients treated with RT and those treated^31,32^ with OT: our results are consistent with those data. However, robotic surgery requires docking time^25^, including positioning the robotic arms, connecting the trocars to robotic arms, and attaching surgical instruments. Therefore, if docking time is excluded, we speculate that the time needed for RT may be shorter than that for OT.

Patient-reported outcome is an important indicator to evaluate postoperative outcome and QoL^5^. NIS, SIS, and VIS have been used objectively to evaluate neck^33^, swallowing, and voice functions, respectively, in endoscopic thyroidectomy and OT, and the SF-36 questionnaire has been used to evaluate the QoL after surgery objectively and accurately^5,34^. However, reports on evaluation of the cosmetic appearance using the SCAR-Q scale after thyroidectomy are lacking^17^. Our results suggest that RT elicited significantly less damage to swallowing actions and the voice than OT. We analyzed this result for four main reasons. First, high-definition three-dimensional imaging systems improve RLN identification in RT. Second, the RLN runs behind or laterally to the true capsule of the thyroid gland, and SMCD can preserve the true capsule behind the thyroid gland. This action increases the safe distance between the instrument and RLN during thyroidectomy, which reduces the risk of complications (e.g. hoarseness and voice changes) caused by nerve damage. Third, robotic surgery is more flexible and precise^25^, allowing for more accurate manipulation between the platysma and strap muscles during flap dissection, which may result in less adhesion between these two layers of muscles. Fourth, a neck incision is not made, so the platysma does not need to be cut, which may result in formation of less scar tissue in RT. Therefore, compared with OT, RT can reduce the risk of complications such as swallowing impairment caused by neck adhesion.

Data from SF-36 suggested that robotic surgery could improve QoL significantly after thyroidectomy. We also found (for the first time) that robotic surgery improved cosmetic results according to the SCAR-Q scale by evaluating incisions postoperatively. However, the score for “body pain” was relatively higher in the RT group than in the OT group. This observation may have been because of the need to establish subcutaneous tunnels in the chest wall, which increased the area of subcutaneous trauma and prolonged postoperative pain in the RT group.

Our study had three main limitations. First, this was a prospective cohort study with a limited level of evidence. Second, not all patients underwent laryngoscopy after surgery, and the rate of RLN injury may have been underestimated. Third, robot-assisted SMCD was implemented only via the UABA approach: whether it can be accomplished via another approach merits further study.

## Conclusions

We summarized the details and key points of robot-assisted SMCD. This is the first prospective cohort study to demonstrate that RT with SMCD can achieve the same level of efficacy and better PG protection than that offered by OT. The postoperative QoL indicators of voice, swallowing, and neck functions were superior when using RT. The latter may become the first-line method for minimally invasive surgery for TC.

## Ethical approval

The study protocol was approved by the Ethics Committee of the First Affiliated Hospital of Army Medical University (Chongqing, China). The present study is registered at www.clinicaltrials.gov (ChiCTR2000033674).

## Consent

Written informed consent was obtained from the patient for publication of this case report and accompanying images. A copy of the written consent is available for review by the Editor-in-Chief of this journal on request.

## Source of funding

This study was supported by Chongqing Municipal Special Project for Technological Innovation and Application Development Project (Application Study of Super-meticulous Capsular Dissection Technique in Robot-Assisted Total Thyroidectomy, No. cstc2019jscx-msxmX0284).

## Author contribution

Q.X.Q.: study design, literature search, data analyses, manuscript drafting, and tables. J.L. and J.M.: literature search and data collection. X.Z.C and J.J.Z.: collection and analyses of data. Q.X.Q, J.L. and J.M. are considered co–first authors. J.J. and Y.Z.: Administrative, technical, material support, or Supervision. L.J.Z.: critical revision of the manuscript. L.J.F.: study design, data analyses, and critical revision of the manuscript. L.J.Z. and L.J.F. had full access to all the data in the study and take responsibility for the integrity of the data and the accuracy of the data analysis.

## Conflicts of interest disclosure

None.

## Research registration unique identifying number (UIN)

The present study is registered at www.clinicaltrials.gov (ChiCTR2000033674).

## Guarantor

Lunjun Fan.

## Provenance and peer review

Not commissioned, externally peer-reviewed.

## Data statement

Supplemental digital content is available for this article. Direct URL citations appear in the printed text and are provided in HTML and PDF versions of this article on the journal website.

## Acknowledgements

The authors also thank all the patients included in this study.
